# Pilot Animal Study on Robotic-Assisted Endovascular Visceral Interventions

**DOI:** 10.1007/s00270-022-03204-0

**Published:** 2022-06-28

**Authors:** Patrick A. Kupczyk, Ulrike I. Attenberger, Carsten Meyer, Julian A. Luetkens, Daniel Kuetting

**Affiliations:** 1 Department of Diagnostic and Interventional Radiology, University Hospital Bonn, Venusberg-Campus 1, 53127 Bonn, Germany; 2 Quantitative Imaging Lab Bonn (QILaB), University Hospital Bonn, Venusberg-Campus 1, 53127 Bonn, Germany

**Keywords:** Robotics, Endovascular, Visceral, Embolization, Stenting

## Abstract

**Purpose:**

To evaluate technical feasibility and safety of common endovascular visceral interventions using a vascular robotic platform through preclinical study.

**Material and Methods:**

The CorPath GRX Robotic System (Corindus Inc, Waltham, Massachusetts) was tested in an anesthetized pig for its ability to navigate various commercially available devices in the abdominal vasculature and to perform routine endovascular visceral procedures. After manually placing a guiding catheter in the celiac trunk, several visceral branches were probed with microcatheters and -wires under robotic assistance, and embolization with liquids (lipiodol), detachable coils and plugs were performed. Furthermore, the origin of the celiac trunk was stented before accessing the left hypogastric artery for pelvic embolization.

**Results:**

All procedures were performed with technical success and without any complications. Navigating the catheters and wires via the steering console proved intuitive. Coil, plug and stent deployment were exclusively controlled by remote with remarkable precision and stability.

**Conclusion:**

Robotic-assisted visceral embolization and stenting as well as pelvic embolization using the CorPath GRX System is feasible and safe. Application of the platform in the abdominal vasculature is demonstrated for the first time. Considering the precision and the potential for reducing the operator’s radiation exposure, further research in this area is highly encouraged to enable translation into clinical practice.

## Introduction

Over the last 20 years, the routine use of robotic systems in medicine has become a clinical reality [1]. Robotic platforms offer the operator the prospect of a high degree of control, while working remotely from a comfortable position [2], minimizing occupational hazards and paving the way for telemedicine and telementoring [3–5]. In terms of robotic-assisted endovascular procedures, first-generation devices required system-dedicated catheters and high-profile sheaths, allowing only for manual microcatheter manipulation and device delivery [6]. Technically refined platforms now enable navigation of off-the-shelf equipment, making them more attractive for advanced applications.

The CorPath GRX (Corindus, Waltham, MA) is FDA-approved for percutaneous coronary and peripheral vascular interventions [7–9]. Given the promising results for neurovascular applications [10, 11], the question arises whether the device is suitable for procedures in similarly delicate and tortuous vessels, such as in the abdomen.

In this pilot study, we investigated the feasibility and safety of performing common endovascular visceral interventions in a porcine model with assistance of the CorPath GRX Robotic System.

## Material and Methods

### Robotic System

**Fig. 1:**
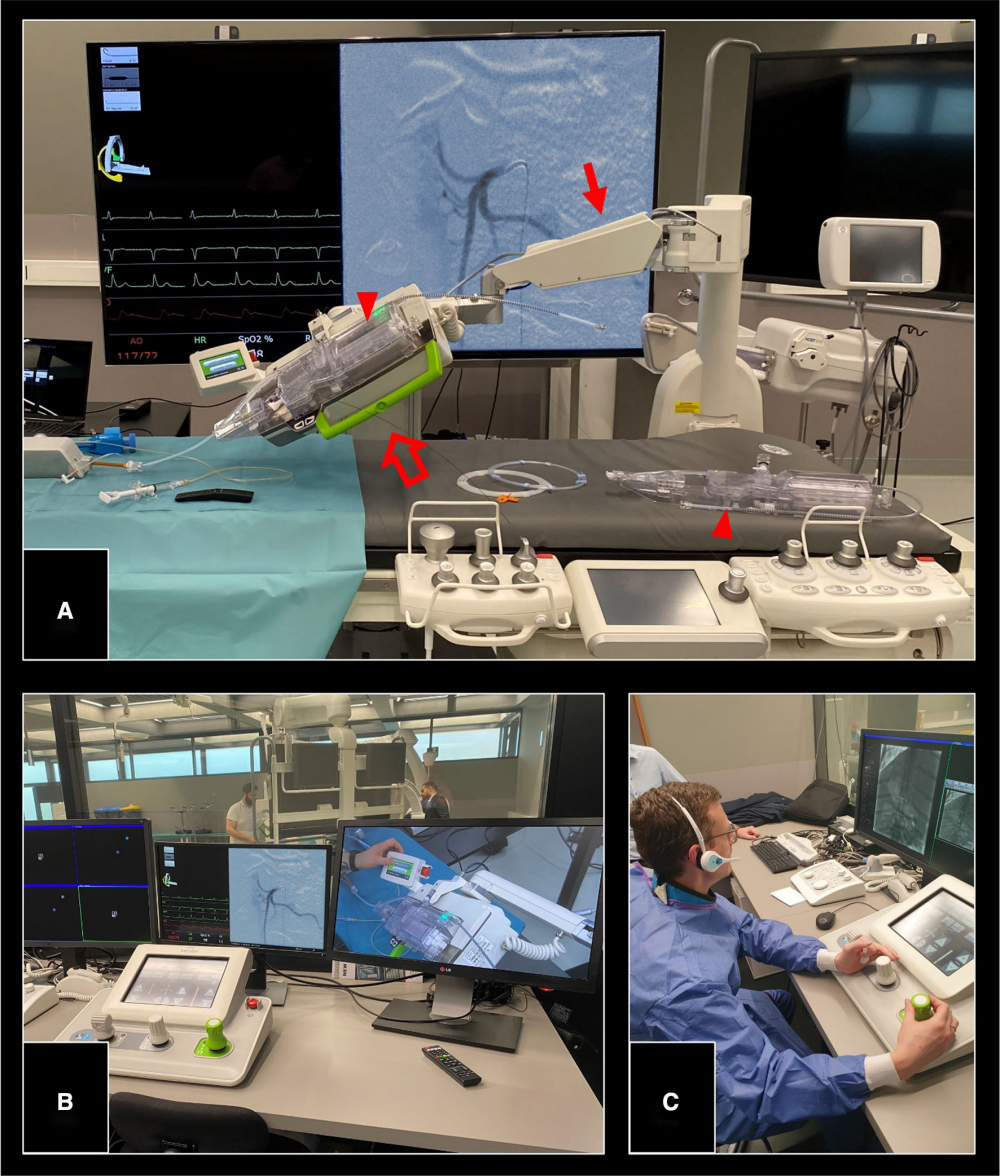
Representative procedure set-up using the CorPath GRX Robotic system. Bedside unit consisting of an articulating arm serving as the device mount (solid arrow), the robotic drive system (hollow arrow) and a single-use sterile cassette (arrowheads) (**A**). Remote radiation-shielded workstation with monitors and controls, the “cockpit” (**B**). Operator navigating mounted catheters, wires and devices via joysticks from the outside while maintaining audiovisual contact to the bedside assistant (**C**)

A CorPath GRX Neurovascular Robotic System (Corindus, Waltham, MA) was used. The principal components are depicted in Fig. 1 and have been described in detail previously [12, 13]. In brief, the platform consists of a bedside unit and a robotic control console. As the clearance of the device is not restricted to a specific vascular territory, this study investigated an on-label use.

### Porcine Model

The intervention was conducted at the ORSI academy (Melle, Belgium) in accordance with the international regulations for the protection of laboratory animals. A single female domestic pig was anesthesized and monitored by veterinarians. Vascular access was obtained in the right common femoral artery under sterile conditions (8F Radifocus Introducer, Terumo, Leuven, Belgium). At the end of the experiment, materials were removed, the vascular access site closed, and the animal humanely euthanized.

### Procedures

Two radiologists (D.K., P.A.K.) with 7 and 5 years of experience in image-guided interventions performed the procedures on an angiography system (Discovery IGS730, GE, Buckinghamshire, UK).

In conventional technique, a 4F selective catheter (Cobra C2, Cordis, Brussels, Belgium) was manually advanced into a stable position in the celiac trunk using a 0.035-inch guidewire (Radifocus Guide Wire M, Terumo) and contrast (iohexol, 300 mg iodine/ml, Accupaque 300, GE). A 2.5F microcatheter (Renegade, Boston Scientific, Marlborough, MA) and a 0.014-inch guidewire (Transend, Boston Scientific) were introduced coaxially and set up in the table-side unit. Using the joysticks in the cockpit, the microcatheter system was successively navigated into both hepatic arteries with occasional assistance of automated steering maneuvers [14]. Embolization with ethiodized oil (Lipiodol, Guerbet, Villepinte, France) was manually performed under fluoroscopy and distribution of the embolization agent verified by conebeam CT.

Moreover, detachable microcoils (Interlock-18, 3 × 60 mm, 4 × 80 mm, Boston Scientific) were advanced by remote, retracted for test purposes and deployed. The stability of the system was tested by deploying an oversized coil in the right hepatic artery, after anchoring it in the gastroduodenal artery.

Following manual intubation of the splenic artery with the Cobra catheter, an Amplatzer Vascular Plug 4 (Abbott, Chicago, ILL) was mounted on the CorPath. Plug positioning and deployment was carried out at the cockpit.

A 7F guiding catheter (Highflow, Cordis) and a 0.014-inch support wire (Hi-Torque Spartacore, Abbott) were then placed in the splenic artery. A balloon-expandable stent (5 × 20 mm Hippocampus, Medtronic, Dublin, Ireland) was introduced, with all three components controlled via the console. After stent advancement, repositioning and catheter retraction with support of another automated maneuver, the “active device fixation” [15], it was manually deployed in the celiac trunk.

Eventually, aortography was followed by manual placement of the selective catheter in the left hypogastric artery. The microcatheter system was connected to the robotic device and guided into the left middle rectal artery, where lipiodol embolization concluded the experiment.

### Outcome Measures

Technical success of the embolization procedures was defined as the angiographically proven complete occlusion of the target vessels. Stenting was considered successful when deployment was complete and accurate, followed by proper opacification of the celiac trunk. Special attention was paid to the occurrence of vascular injury and nontarget embolization.

## Results

**Fig. 2:**
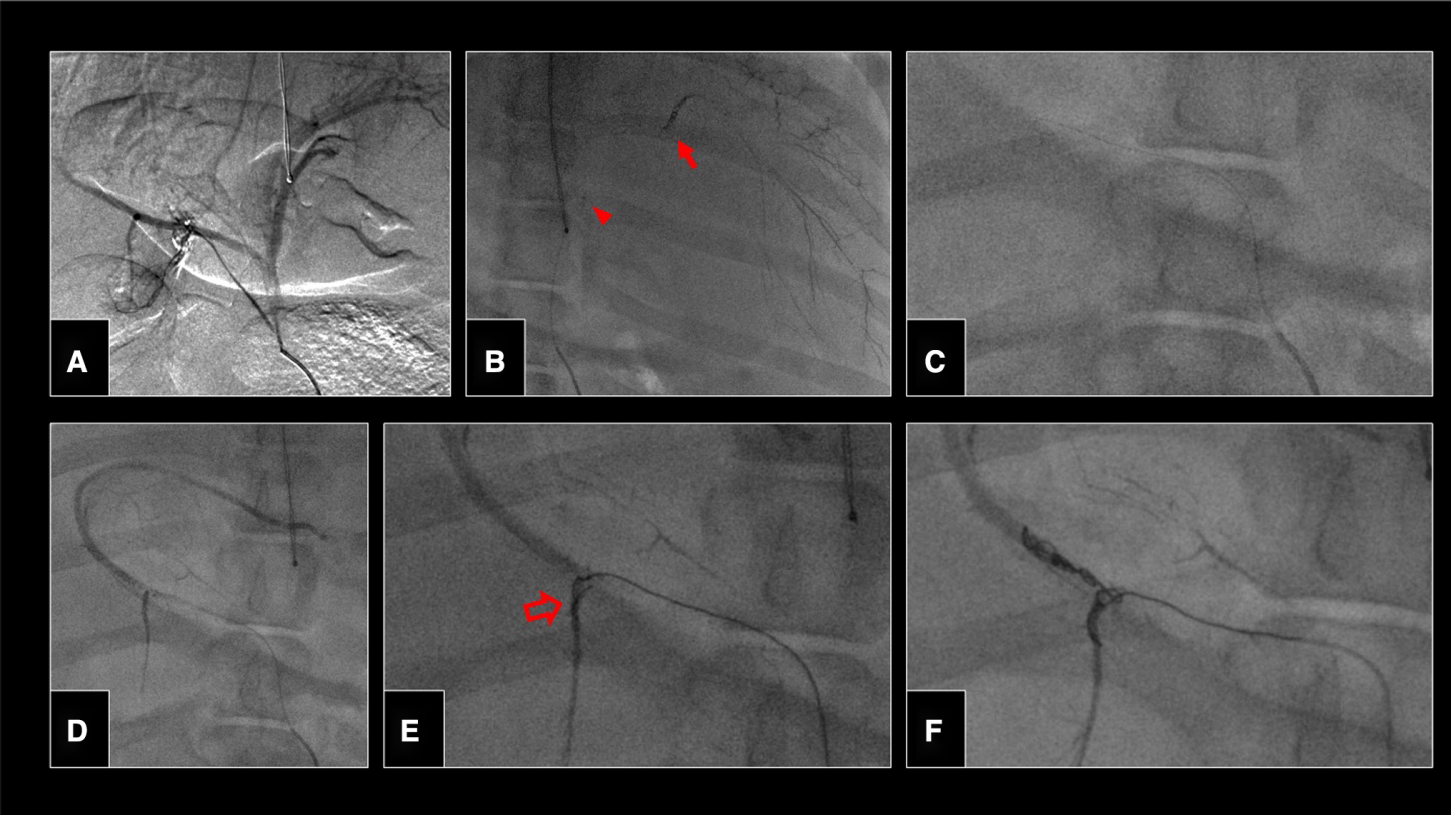
Angiography of the celiac trunk (**A**). Bland embolization of the left hepatic artery with ethiodized oil (Lipiodol, Guerbet, Villepinte, France) following robotic-assisted superselective catheterization. Note the position of the microcatheter tip (red arrowhead) and a previously deployed coil further distally (red solid arrow) (**B**). Embolization of the right hepatic artery (**D**) with a mechanically detachable microcoil (red hollow arrow; Interlock-18, 4 × 80 mm, Boston Scientific, Marlborough, MA) in anchor technique (**E**) deployed remotely via the robotic unit (**F**)

**Fig. 3:**
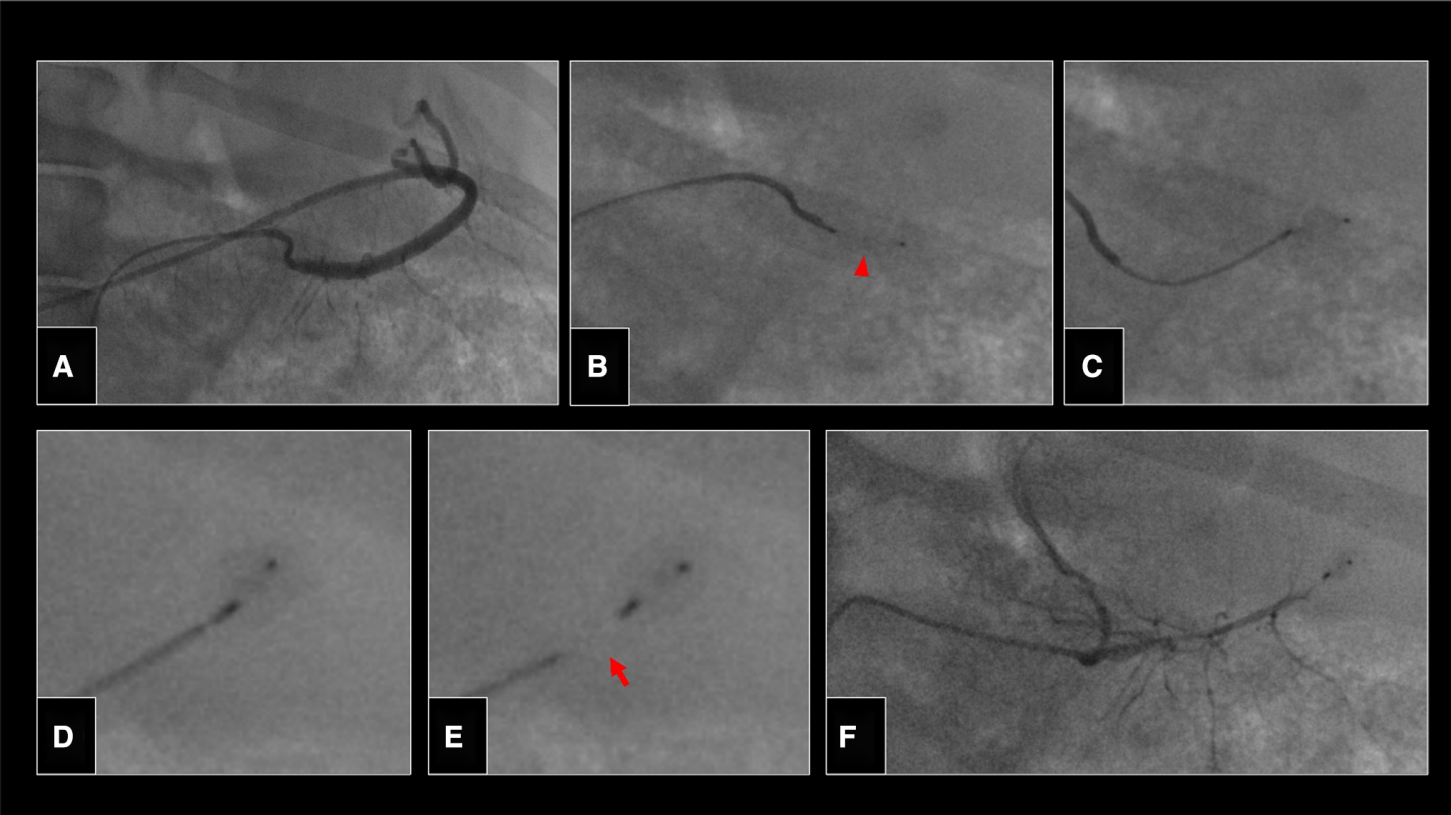
Angiography of the splenic artery after robotic-assisted intubation with a 4F Cobra catheter (**A**). Robotic-assisted placement of an Amplatzer Vascular Plug 4 (red arrowhead; Abbott, Chicago, ILL) (**B**, **C**) in the splenic artery. Plug detachment (**D**, **E**) was completely performed via the robotic unit. Angiography following plug deployment showing complete occlusion of the splenic artery (**F**)

Although both interventionalists had no prior experience with the robotic platform, superselective catheterization of the different vascular territories using the remote controls proved intuitive with a steep learning curve. The microcatheter was consistently navigated to the visceral target vessels within three minutes. Thereby, control angiographies showed no signs of dissection or perforation. There was neither dislocation of the microcatheter tip during lipiodol delivery nor nontarget embolization. Robotic-assisted delivery, retraction and deployment of microcoils in small-caliber vessels was feasible and precise; the anchor technique was realized without catheter recoil or the need for repositioning (Fig. 2). Controlled advancement, repositioning and detachment of a vascular plug via the cockpit were demonstrated in the splenic artery (Fig. 3). Vascular occlusion was achieved in all treated territories.

**Fig. 4:**
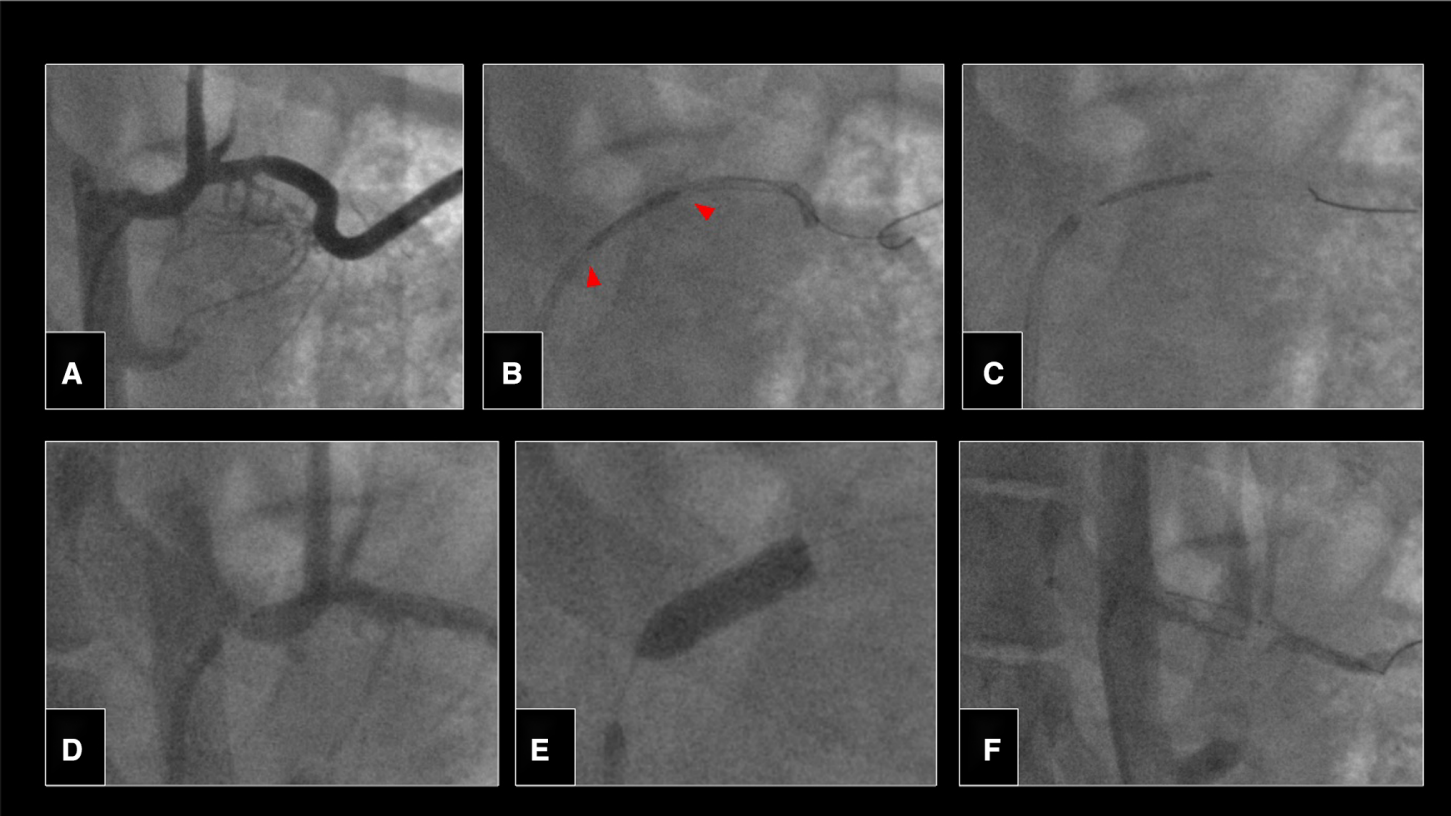
Angiography of the celiac trunk via a 7F guiding catheter (Highflow, Cordis) (**A**). Robotic-assisted advancement of a balloon-expandable bare metal stent (5 × 20 mm Hippocampus, Medtronic, Dublin, Ireland) (red arrowheads) into the celiac trunk (**B**). Retraction of the guiding catheter using the “active device fixation” feature of the CorPath GRX keeping the advanced stent in place (**C**). After robotic-assisted positioning of the stent and control angiography (**D**), manual balloon inflation to expand the stent (**E**). Control angiography showing satisfactory position of the stent (**F**)

**Fig. 5:**
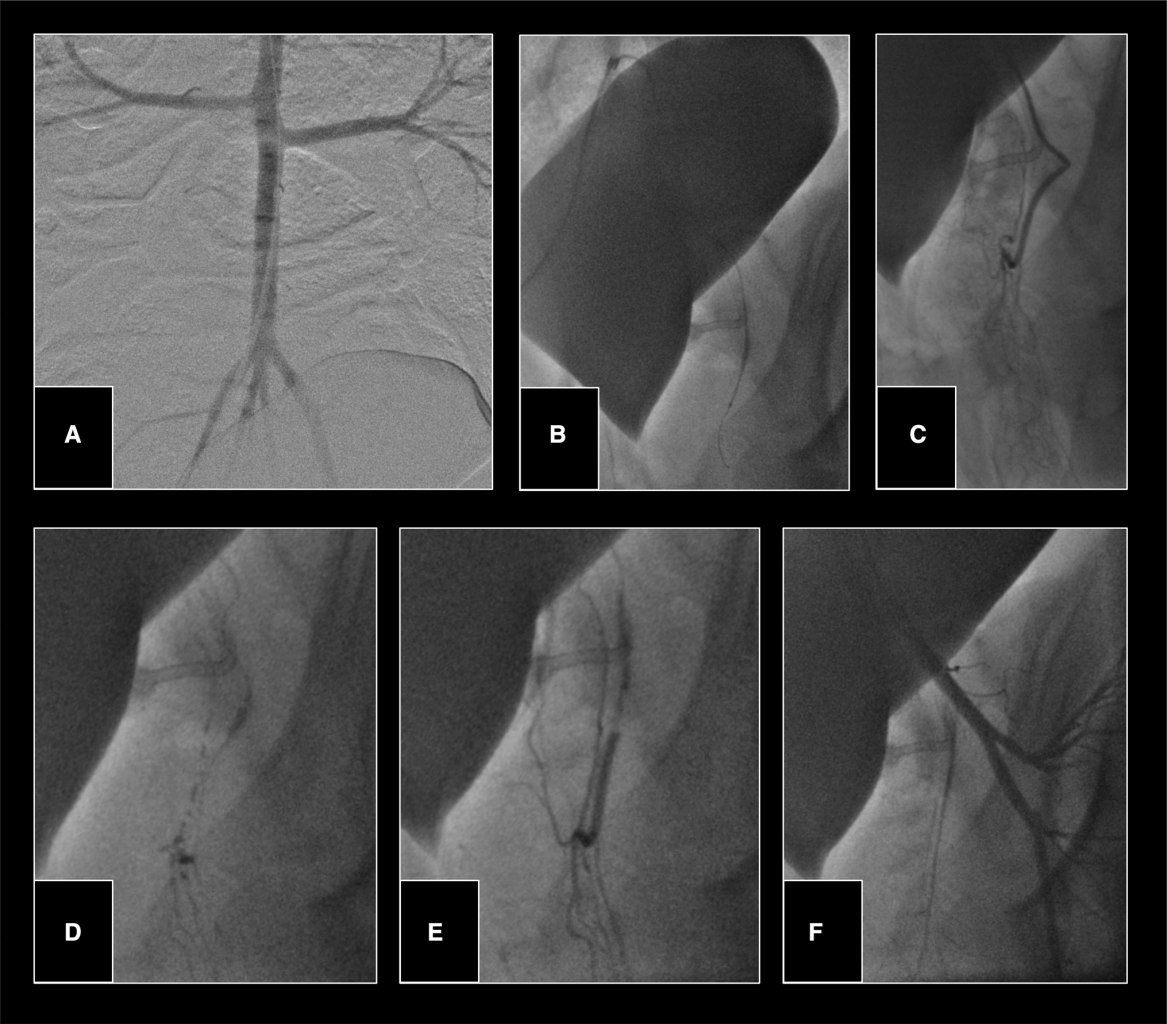
Aortography via the 8F introducer sheath (**A**). Cross over catheterization followed by robotic-assisted superselective catheterization of the left middle rectal artery (**B**, **C**). Manual lipiodol application at this site (**D**), until stasis was seen (**E**). Final angiography showing satisfactory occlusion of the middle rectal artery (**F**)

Under simultaneous control over the guiding catheter, stent and support wire, a stent was accurately deployed in the celiac trunk (Fig. 4). Finally, the microcatheter system was successfully navigated in the pelvic vasculature enabling superselective embolization of a visceral branch (Fig. 5).

## Discussion

Robotic-assisted endovascular visceral interventions of varying complexity were successfully performed in a pig using the CorPath GRX Robotic System.

Of note is the ease and precision with which the untrained operators could remotely navigate the microcatheter system in a clinically acceptable time. Thus, the reduced exposure time and the redundancy of protective equipment may minimize radiation-related and other occupational hazards to the operator [16–20]. This may prove particularly beneficial in visceral procedures, where the often cumbersome cannulation of small-caliber and tortuous vessels can contribute to operator fatigue and poor outcomes [21–23]. The observed catheter and device stability is fundamental for avoiding potentially organ- or life-threatening nontarget-embolizations and makes typical visceral embolization procedures (e.g., TACE, TARE, splenic artery embolization) an ideal application for the platform. Eventually, as visceral stenting can be challenging due to coordination difficulties and lack of stability, the simultaneous control over guiding catheter, wire and stent could help to overcome these drawbacks.

However, there remain several limitations to this innovative technology. First, device setup and exchanging interventional equipment requires specialized training; the expected time expenditure does not currently justify its use in emergency settings. Secondly, the lack of haptic feedback raises concerns about accidental vascular injury; however, novel technological developments address this issue by introducing force feedback and collision detection mechanisms [24–26]. Lastly, compatibility is restricted to monorail systems, which have less pushability with smaller diameters and lengths.

As we performed the procedures on a single animal, generalization of our observations regarding safety and efficacy of performing endovascular visceral interventions with the CorPath requires a larger sample size. In addition, without comparison to manually performed procedures, statements about clinical benefits remain hypothetical. However, this was not the subject of this feasibility study; rather, it was intended to provide a first impression of the possibilities and limitations of this cutting-edge technology in visceral applications.

## Conclusion

Robotic-assisted endovascular visceral interventions are feasible with the CorPath GRX platform. The system combines a considerable level of control over customary interventional equipment in complex vascular territories with the potential benefits of reducing occupational hazards to the operator and improving clinical outcomes. To extend regulatory approval to visceral applications, further research on efficacy and safety is highly encouraged.
